# From soil to plant, the journey of P through trophic relationships and ectomycorrhizal association

**DOI:** 10.3389/fpls.2014.00548

**Published:** 2014-10-15

**Authors:** Adeline Becquer, Jean Trap, Usman Irshad, Muhammad A. Ali, Plassard Claude

**Affiliations:** ^1^UMR Eco&Sols, Institut National de la Recherche AgronomiqueMontpellier, France; ^2^UMR Eco&Sols, Institut de Recherche pour le DéveloppementMontpellier, France; ^3^Department of Environmental Sciences, COMSATS Institute of Information TechnologyAbbottabad, Pakistan; ^4^Department of Soil Science, Bahauddin Zakariya UniversityMultan, Pakistan

**Keywords:** phosphate, tree P nutrition, bacterial grazers, ectomycorrhizal association, phosphate transport systems

## Abstract

Phosphorus (P) is essential for plant growth and productivity. It is one of the most limiting macronutrients in soil because it is mainly present as unavailable, bound P whereas plants can only use unbound, inorganic phosphate (Pi), which is found in very low concentrations in soil solution. Some ectomycorrhizal fungi are able to release organic compounds (organic anions or phosphatases) to mobilize unavailable P. Recent studies suggest that bacteria play a major role in the mineralization of nutrients such as P through trophic relationships as they can produce specific phosphatases such as phytases to degrade phytate, the main form of soil organic P. Bacteria are also more effective than other microorganisms or plants at immobilizing free Pi. Therefore, bacterial grazing by grazers, such as nematodes, could release Pi locked in bacterial biomass. Free Pi may be taken up by ectomycorrhizal fungus by specific phosphate transporters and transferred to the plant by mechanisms that have not yet been identified. This mini-review aims to follow the phosphate pathway to understand the ecological and molecular mechanisms responsible for transfer of phosphate from the soil to the plant, to improve plant P nutrition.

## INTRODUCTION

Phosphorus (P) is an essential element for plant growth and productivity. P is a component of nucleic acids, phospholipids, and ATP and, as such, is involved in controlling enzyme reactions and the regulation of the metabolic pathway (Schachtman, 1998; Raghothama, 1999; Vance et al., 2003). Plants can only take up P as free phosphate ions, H_2_PO_4_^-^ and HPO_4_^2-^(Pi). However, concentrations of free Pi in soil solution are generally low, around 1–10 μM (Hinsinger, 2001), owing to its strong affinity for combining with cations and clays, leading to the formation of insoluble P complexes that are unavailable for plants (Hinsinger, 2001). P is, therefore, one of the most limiting macronutrients for plant growth (Raghothama, 1999) and productivity (Batjes, 1997) in many terrestrial ecosystems. However, plants are involved in complex ecological interactions, especially through symbiotic mycorrhizal association, allowing them to meet their P requirements (Bucher, 2007; Javot et al., 2007; Lambers et al., 2008; Plassard and Dell, 2010).

In forest ecosystems, particularly in temperate and boreal biomes, 95% of trees establish a mutualistic ectomycorrhizal (ECM) symbiosis with fungal symbionts (Smith and Read, 2008). The ECM fungus forms a soil-fungus interface outside the roots of the host plant with a hyphal sheath around short lateral roots and extra-radical hyphae growing from the sheath. The hyphae explore a large volume of soil not accessible to roots and allow the translocation of nutrients and water to the host plant in exchange for sugar (Nehls et al., 2010). The nutritional exchanges between fungus and host occur in the Hartig net located at the interface between the root cortical cells and the fungal hyphae. The formation of symbiotic structures with ECM fungi is considered to be the most widespread means of increasing P acquisition by trees (Chalot et al., 2002; Torres Aquino and Plassard, 2004; Smith and Read, 2008).

This mini-review considers the outward journey of P, from soil to tree through ECM association. It discusses the mechanisms by which the fungus mobilizes poorly available organic P (Po) sources such as phytate and takes up Pi at the soil–fungus interface. It then summarizes current knowledge of the fungus–plant interface and suggests hypotheses concerning the transfer of P from the fungus to the plant.

## ROLE OF ECTOMYCORRHIZAL FUNGI AT THE SOIL-FUNGUS INTERFACE

### ECM AND PHYTATE MOBILIZATION

**FIGURE 1:**
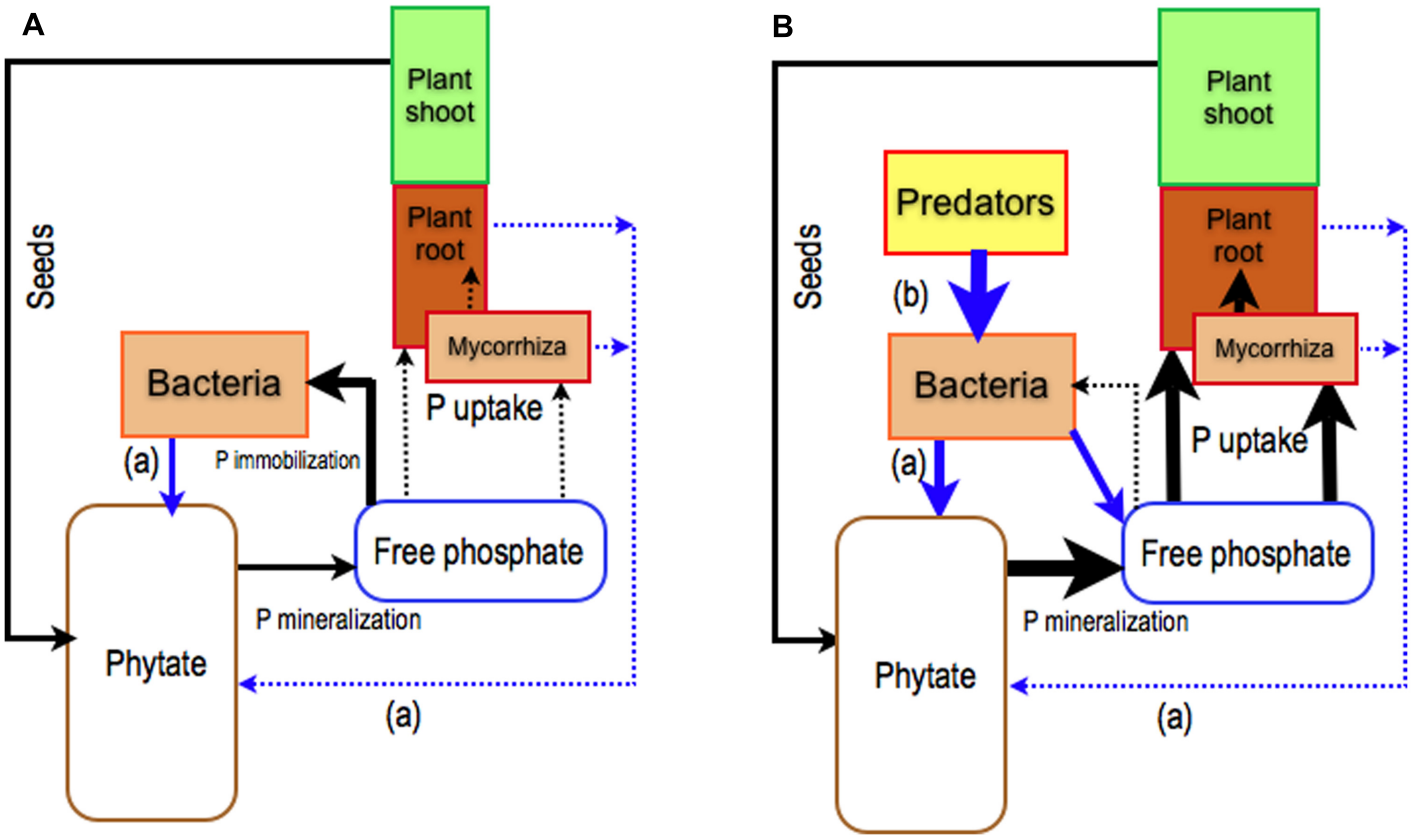
**Role of plants and their mycorrhizal symbionts together with rhizosphere bacterial populations on the use of soil phytate.** Phytate is the main pool of organic phosphorus (Po) in soil ( Turner et al., 2002) that is filled by phytate from ungerminated seeds. To be used by plants, phytate must be hydrolyzed by specialized enzymes called phytases. **(A)** The capacity of roots and ECM fungi to release phytases in the rhizosphere is very low (Richardson et al., 2007) whereas some bacteria have a great ability to produce phytase and to mineralize phytate (Jorquera et al., 2008a,b) for their own. This will result in bacterial P immobilization at the expense of plants and ECM fungi. **(B)** To improve P nutrition of plants alone or with ECM fungi, P from phytate locked in bacteria has to be released through the grazing activity of microfauna, such as bacterial grazer nematodes (Irshad et al., 2012). Black arrows: P fluxes, blue arrows: biological controls, (a) release of phytase, (b) grazing activity.

A large proportion of P in forest soils is found as Po compounds (Tibbett, 2002). Most Po is in the form of phosphate esters (C-O-P bonds) such as phosphate monoesters (e.g., sugar–phosphates) and phosphate diesters (nucleic acids and phospholipids; Turner, 2008). Rennenberg and Herschbach (2013) suggested that ECM fungi might absorb Po as a whole molecule. The identification of three genes encoding glycerophosphoinositol transporters in the *Hebeloma cylindrosporum* genome (JGI project list) supports this hypothesis but the activity of these transporters has not yet been established. Furthermore, it is generally accepted that, in order to be used by plants and microorganisms, phosphate groups must be released from the ester bond by phosphatase enzymes (Plassard and Dell, 2010). Of soil Po compounds, phytate (*myo-inositol hexakisphosphate*), a form of inositol phosphate, is particularly interesting as a potential source of P for plants, because it is found in many ecosystems, including forest ecosystems (Turner et al., 2002). Phytate is a form of P reserve in seeds (Raboy, 2007) and it is hydrolyzed during germination by intracellular plant phytases to supply Pi to young seedlings. However, if the seeds do not germinate, their phytate content will fill the pool of soil phytate (**Figure 1**). To be used by plants and microorganisms, phosphate groups of phytate must be released by specialized enzymes (phytases). The efficiency of organisms in mobilizing phytate in the soil solution relies on their ability to produce phytases in the external medium or at least in the cell wall space. To date, plants grown in axenic conditions have been shown to have very poor capacity to use phytate as the sole source of P (Hayes et al., 2000; Richardson et al., 2000, 2001a,b), suggesting that they have little or no capacity for releasing phytase into the external medium (**Figure 1**). The capacity of ECM fungi to release phytase is still a matter of debate: some studies have reported that ECM basidiomycetes have a high capacity (Antibus et al., 1992; McElhinney and Mitchell, 1993), no capacity (Mousain et al., 1988) or a very low capacity (Mousain et al., 1988; Louche et al., 2010) to produce phytase in axenic cultures.

Current knowledge suggests that ECM fungi on their own are not the best symbionts for improving plant nutrition using phytate as the sole source of P (Richardson et al., 2007; Plassard et al., 2011; **Figure 1A**). Another strategy that has been little studied until now relies on the exploitation of the interactions between plants, ECM fungi, bacteria, and their grazers within the rhizosphere (food web relationships) combined with the capacity of bacteria to degrade phytate (**Figure 1B**).

### ROLES OF RHIZOSPHERE TROPHIC INTERACTIONS

Unlike ECM fungi, bacteria inhabiting the plant rhizosphere are able to mineralize phytate *in vitro* (Jorquera et al., 2008a,b; Maougal et al., 2014). Several studies have shown that inoculating plants with these bacteria, in sterile conditions, improves plant access to P from phytate (Richardson and Hadobas, 1997; Hayes et al., 2000; Richardson et al., 2001b). While the plants provide carbon, the bacteria mineralize Po and increase the available P pool. However, bacteria are more competitive than plants and ectomycorrhizal fungi to take up P released by phytase (Irshad et al., 2012). In consequence, a large fraction of P released from phytate is immobilized and locked in the soil bacterial biomass (**Figure 1A**). It could, therefore, be possible that bacterial grazers significantly improve plant P nutrition through re-mineralization of the microbial P pool (soil microbial loop), and reduction of the competition between plants and bacteria for Pi (Clarholm, 1985; **Figure 1B**). Nevertheless, studies focusing on protozoa (Coleman et al., 1977; Cole et al., 1978; Griffiths, 1986; Darbyshire et al., 1994; Bonkowski et al., 2001) and nematodes (Anderson et al., 1978; Griffiths, 1986; Bardgett and Chan, 1999; Irshad et al., 2011, 2012) reported that bacterial grazers have either no effect (Griffiths, 1986), a short-term increase (Anderson et al., 1978; Darbyshire et al., 1994; Djigal et al., 2004) or a significant increase (Coleman et al., 1977; Cole et al., 1978) on soil P mineralization with obvious consequences on plant P nutrition (Herdler et al., 2008; Irshad et al., 2012). These contradictory results were put forward without any clear identification of the ecological factors driving the efficiency of bactivorous-induced P mineralization. More specifically, the study carried out by Irshad et al. (2012), with phytate as the sole source of P, showed that the presence of both bacterial-feeder nematodes (*Rhabditis sp.*) and *Bacillus subtilis* increased the net amount of P in *Pinus pinaster* seedlings (**Figure 1B**). A possible mechanism involved in these patterns lies in the ability of grazers to increase bacterial metabolism and, probably, phytase production (**Figure 1B**). It would, therefore, be interesting to study the expression of two main classes of bacterial phytase genes, the histidine acid phytases (HAP), and the β-Propeller phytase (BPP) (Mullaney and Ullah, 2003) when bacteria are in the presence of their grazers. It may be supposed that predation has two synergistic effects; (i) grazers may cause the overexpression of bacterial HAP and/or BPP and increase the mineralization of phytate and (ii) grazers may increase phosphate availability by the release of P from the microbial biomass (**Figure 1B**).

However, it is not clear how the presence of ECM fungi affects this positive trophic P pathway. The experimental study conducted by Irshad et al. (2012) with *P. pinaster* showed that the presence of the ECM fungi (*H. cylindrosporum*) did not alter the positive trophic effect on plant P nutrition from phytate. This was probably due to experimental conditions used by the authors, which used agarose medium rather than soil. ECM fungi could be expected to have a positive effect by increasing the soluble mineral P uptake by the host from the additional P released by nematodes and subsequently locked in the soil matrix (Plassard and Dell, 2010). The role of ECM fungi remains unclear and further studies are clearly needed.

### ECM AND INORGANIC PHOSPHORUS ACQUISITION

After mineralization of phytate and other Po compounds, the phosphate released must be absorbed by plants and mainly by ECM fungi, which are more efficient than the roots. Van Tichelen and Colpaert (2000) showed that ECM fungi significantly increased the phosphate uptake capacity of pine roots. As discussed above, the mycorrhizal basidiomycete produces an extra-radical mycelium that is able to explore the soil away from the root, significantly increasing the volume of soil exploited by plants with ECM fungi. It has been shown that such exploration was responsible for the major fraction of P uptake by plants (Torres Aquino and Plassard, 2004).

**FIGURE 2:**
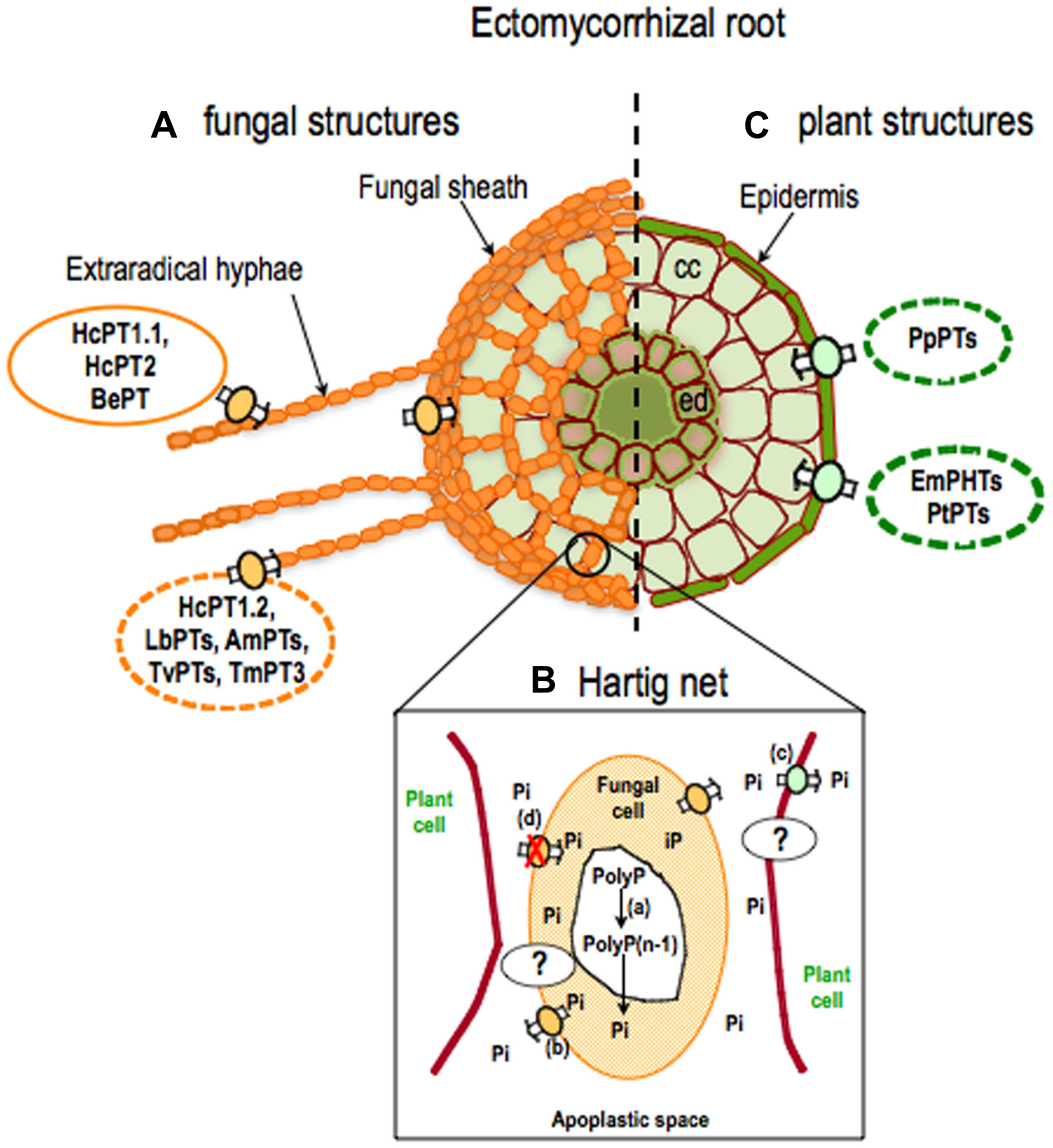
**Current knowledge about phosphate transporters in ectomycorrhizal roots.** In ectomycorrhizal (ECM) roots, the fungus forms extraradical hyphae and a fungal sheath outside the root **(A)** and the Hartig net surrounding root cells **(B)** hiding epidermal cells and cortical (cc) cells **(C)**. **(A)** In fungal cells, the uptake of Pi occurs mostly through Pht1 phosphate transporters. To date, only *HcPT1.1*, *HcPT2* ( Tatry et al., 2009), and *BePT* ( Wang et al., 2014) genes have been characterized by heterologous expression in yeasts. Genomics and transcriptomic data suggest that other transporters may play a role in phosphate uptake (e.g., *HcPT1.2*, *LbPTs*, *AmPTs*, *TvPTs*, *TmPT3* (Pht2; Casieri et al., 2013). **(B)** In the Hartig net, fungal and plant cells have a common apoplastic space with no direct symplastic communication. It is hypothesized that the hydrolysis (a) of polyphosphate (PolyP) increases Pi concentration in the cytosol of the fungus. Up to now, the molecular mechanisms sustaining P eﬄux from the fungus (b) to the apoplast and P influx (c) from the apoplast to the plant cell have not been identified. It is also hypothesized that fungal P transporters may not be functioning (d). **(C)** In plant cells, phosphate ions enter through plant P transporters. Little is known about plant transporters responsible for Pi acquisition in ECM roots. Only phosphate transporters from *Populus trichocarpa* (PtPTs; Loth-Pereda et al., 2011) and *Eucalyptus marginata* (EmPhts; Kariman et al., 2014) have so far been identified. Transcriptomic data for *Pinus pinaster* (Canales et al., 2013) showed putative encoding sequences for phosphate transporters (PpPTs). Full lines indicate transport systems whose capability in phosphate transport has been verified by heterologous expression in yeast. Dotted lines indicate transport systems whose involvement in phosphate transport during mycorrhizal symbioses is suggested by genomic or transcriptomic data. ed: endodermal cells. Hc: *Hebeloma cylindrosporum*, Be: *Boletus edulis*, Tm: *Tuber melanosporum,* Am: *Amanita muscaria*, Lb: *Laccaria bicolor*, Tv: *Tricholoma vaccinum*, Pt: *Populus trichocarpa*, Em: *Eucalyptus marginata*, Pp: *Pinus pinaster.*

The acquisition of free phosphate by ECM fungi occurs through a plasma membrane phosphate transporter (**Figure 2A**). The first putative Pi transporter gene from an ECM fungal species (Kothe et al., 2002) was identified based on homology with the yeast Pi transporter *PHO84* (Bun-ya et al., 1991). More recently, many others have been found in the genomes of five ECM fungi (JGI Genome Portal, Casieri et al., 2013). Most ECM fungi have three to five putative phosphate transporter genes that belong to the *Pht1* subfamily (Karandashov and Bucher, 2005; phosphate/H^+^ transporters). However, the phosphate transporter encoded by the *TmPT3* gene was classified as a phosphate/Na^+^ transporter (*Pht2*). This type of transporter has first been identified in the yeast, *Saccharomyces cerevisiae* (Martinez and Persson, 1998). These results suggest that the efficiency of phosphate uptake into ECM fungal cells could rely mostly upon the external pH. Of all phosphate transporters so far identified in ECM fungi, only HcPT1.1, HcPT2, and BePT have been characterized by heterologous expression in yeast (Tatry et al., 2009; Wang et al., 2014). *HcPT1.1* and *HcPT2* were expressed in *H. cylindrosporum* alone or associated with its natural host plant, *P. pinaster*, grown in low or high P conditions. However, the transporters respond in different ways to the external Pi concentration. *HcPT1.1* transcripts were up-regulated in fungal cells exposed to phosphate starvation in solution or to low phosphate availability in soil such as phosphate transporters of the ECM fungus *Tricholoma spp.* (Kothe et al., 2002) and *Boletus edulis* (Wang et al., 2014), whereas the transcripts levels of *HcPT2* were less dependent on the external P concentration (Tatry et al., 2009). The expression patterns of these two transporters (Tatry et al., 2009) and the immunolocalization of *HcPT1.1* (Garcia et al., 2013) indicate that they are found in extraradical hyphae (**Figure 2**). *H. cylindrosporum* might use *HcPT1.1* to mediate Pi uptake in phosphate starvation conditions and *HcPT2* when soil P availability is high (Tatry et al., 2009).

### PHOSPHATE TRANSLOCATION FROM THE EXTRAMATRICIAL MYCELIA TO THE HARTIG NET

Once absorbed, P is transferred via the extra-radical mycelium to the ECM roots. This has been demonstrated using ^32^P labeling and pulse chase experiments in simple laboratory systems where the distance of translocation did not exceed 40 cm (Finlay and Read, 1986; Timonen et al., 1996). For forests, it is accepted that this range can be much higher (Anderson and Cairney, 2007). The first studies on P translocation reported that ECM hyphae contain a tubular vacuole system (Ashford and Allaway, 2002). Although there is no direct evidence that these vacuoles interconnected by smaller membrane tubules could be involved in P transport, Ashford and Allaway (2002) showed movement of fluorescent probes in this vacuolar system and that ECM fungal vacuoles hold substantial amounts of P mainly in the form of polyphosphates. In parallel, mathematical models suggested that this network was only responsible for short distance P translocation at the mm to cm scale (Darrah et al., 2006; Fricker et al., 2008). Other mechanisms could be involved in longer distance transport but there is still little evidence of this (Cairney, 2011).

## UNDERSTANDING THE FUNGUS–PLANT INTERFACE

### FROM THE FUNGAL CELL TO THE HOST CELL

Interactions between ECM fungi and plants are based on the bidirectional transfer of carbohydrates and nutrients, such as P, across an interface (Bücking and Heyser, 2001). For a long time, studies have demonstrated the transfer of P, mainly as inorganic orthophosphate, from the ECM fungus to the plant (Harley and Loughman, 1963; Skinner and Bowen, 1974; Finlay and Read, 1986). Bücking and Heyser (2001) showed, by microautoradiographic studies following ^33^P, that Pi accumulated rapidly in the ECM sheath and was slowly translocated off the Hartig net to the cortical cells. Because there is no direct symplastic continuity between the ECM fungus and the roots, Pi has to move into the interfacial apoplast before it can be absorbed by the plant (Peterson and Bonfante, 1994; **Figure 2B**). The molecular mechanisms of Pi transport across the mycorrhizal interface have not yet been determined for mycorrhizal symbioses (Harrison, 1999; Plassard and Dell, 2010; Smith and Smith, 2011; **Figure 2B**).

Nutrient transfer models generally involve (1) the passive eﬄux of phosphate and carbohydrates through the fungal and plant plasma membranes into the interfacial apoplasm and (2) the active absorption of nutrients by both symbionts driven by an H^+^-ATPase (Smith and Smith, 2011). However, the net loss of P from ECM fungi in pure culture is normally regarded as slight (Cairney and Smith, 1993a). There must be specific conditions favoring the eﬄux of phosphate from the fungus at the fungus-root interface in ectomycorrhizas in order to ensure that the transfer of P is sufficiently large to meet the host plant demand (Smith and Smith, 2011). It has been suggested that passive Pi flux across the fungal plasma membrane is due to low Pi concentration in the apoplast at the fungus-root interface relative to the cytoplasm (Smith et al., 1994). As suggested for arbuscular mycorrhizal (AM) associations (Solaiman and Saito, 2001), this gradient could be the result of polyphosphate degradation in the fungal cytosol (Clarkson, 1985) and the efficient phosphate uptake across the plant plasma membrane through phosphate transporters (Bucher, 2007; Javot et al., 2007). Moreover, P eﬄux from free-living mycelia of ECM fungi has been shown to be clearly affected by an extracellular supply of cations, particularly K^+^ and Na^+^ and carbohydrates (Cairney and Smith, 1993b; Bücking, 2004). While these observations are derived from mycelia in axenic culture, they provide strong indirect evidence that the eﬄux may be influenced by the chemical environment of the zone of exchange localized in the Hartig net.

Alternatively, the output of Pi from the ECM fungus toward the common apoplasm could be an active mechanism involving phosphate transporters whose presence and/or activity is regulated, at least partly, by host demand (Cairney and Smith, 1992). Genome sequencing of *H. cylindrosporum* has identified three phosphate transporters which have been characterized in yeast as phosphate influx transporters (Tatry et al., 2009). Phosphate eﬄux could be provided by one of these carriers, able to input and output Pi depending on specific conditions. The yeast high affinity phosphate transporter (PHO84) is able to transport phosphate bidirectionally, depending on the pH gradient across the plasma membrane (Fristedt et al., 1996). The phosphate eﬄux could also be mediated by another transport system, as yet unidentified, specifically responsible for phosphate eﬄux at the fungus-root interface (**Figure 2B**).

### THE RELEASE OF P TO THE PLANT

Contrary to AM symbiosis (see Bucher, 2007 for review), little is still known about plant transporters responsible for Pi acquisition in the Hartig net of roots with ECM fungi (**Figure 2B**). The studies by Loth-Pereda et al. (2011) and Kariman et al. (2014) are the first to provide details of the regulation of plant gene expression that may be involved in phosphate uptake by root cells (**Figures 2B,C**). Loth-Pereda et al. (2011) showed that *Populus* colonization by both AM and ECM fungi led to the up-regulation of two *Pht1* transporters, *PtPT9*, and *PtPT12*. These genes are also up-regulated in Pi-depleted media. This suggests that these two genes could be involved in plant Pi uptake in the Hartig net (**Figure 2B**) and/or in Pi acquisition from soil solution (**Figure 2C**). Other plant *Pht1* gene products were down-regulated in ECM symbiosis (Loth-Pereda et al., 2011; Kariman et al., 2014; **Figure 2C**). This will probably result in a reduction in Pi absorption via the direct (root) pathway toward a mycorrhizal pathway as documented for AM symbiosis (Smith and Smith, 2011). However, it is not still clear whether the reduced expression of *Pht1* genes in mycorrhizal roots is triggered by improved P nutrition of plants or whether it is a symbiotic response (Javot et al., 2007). Furthermore, no *Pht1* genes are specifically induced during ECM development as observed in AM symbiosis in woody plants (Loth-Pereda et al., 2011) and herbaceous species (Harrison et al., 2002; Bucher, 2007).

## CONCLUSION

The molecular mechanisms sustaining P fluxes from ECM fungi to root cells in the Hartig net have not yet been explained. This lack of knowledge limits our ability to improve P-utilization efficiency in forest ecosystems. Large scale sequencing of fungal (Martin et al., 2011) and tree (Mackay et al., 2012) genomes will provide candidate genes that may be involved in these P exchanges. Using the genetic transformation methods available for ECM fungi (Combier et al., 2003; Kemppainen et al., 2005; Rodríguez-Tovar et al., 2005; Garcia et al., 2014) and trees (e.g., *P. pinaster,* Alvarez and Ordás, 2013) it will then be possible to study these candidates and determine their actual role in ECM symbiosis.

## Conflict of Interest Statement

The authors declare that the research was conducted in the absence of any commercial or financial relationships that could be construed as a potential conflict of interest.
